# Identification of QTLs associated with *opaque2* modifiers influencing kernel opacity, kernel hardness, and tryptophan content in quality protein maize

**DOI:** 10.3389/fpls.2025.1553512

**Published:** 2025-05-21

**Authors:** Diksha Jasrotia, Sushil Kumar, Yashmeet Kaur, Abhijit Kumar Das, Alla Singh, Dharam Paul, Shanu Shukla, Priti Sharma, Sujay Rakshit, Ramesh Kumar

**Affiliations:** ^1^ Plant Breeding and Genetics, Punjab, Agricultural University, Ludhiana, Punjab, India; ^2^ Indian Council of Agricultural Research (ICAR)-Indian Institute of Maize Research, Ludhiana, Punjab, India; ^3^ Department of Agricultural Botany, Mahatma Phule Krishi Vidyapeeth, Rahuri, Maharashtra, India; ^4^ Indian Council of Agricultural Research (ICAR)-Indian Institute of Agricultural Biotechnology, Ranchi, Jharkhand, India

**Keywords:** QTL mapping, quality protein maize (QPM), *o2* endosperm modifiers, kernel opacity, kernel hardness, tryptophan content

## Abstract

Lysine and tryptophan, two essential amino acids, are generally deficient in normal maize but enriched in *opaque2* (*o2*) mutants. However, these *o2* mutants are linked to undesirable effects like soft endosperm and yield loss. To circumvent this, researchers introgressed *o2 modifiers* (*Mo2*s) into mutant maize and developed Quality Protein Maize (QPM). This study identifies genomic regions linked to *Mo2* governing kernel hardness, opacity, and tryptophan content. Two QPM lines (DQL 2104–1 and DQL 2034), contrasting for these traits, were crossed to develop a 138 F_2_ and 109 F_2:3_ mapping population. Genotyping with 141 informative SSR markers resulted in 2417.01 cM genetic map with an average marker distance of 20.66 cM between markers. Inclusive composite interval mapping (ICIM) detected 11 QTLs across six different chromosomes: seven QTLs for kernel opacity (chromosomes 1, 2, 4, 7), three for hardness (chromosomes 7, 8, 9), and one for tryptophan (chromosome 9). These QTLs co-localized with candidate genes (*opaque1, opaque11, floury1, floury2, floury4, mucronate1*, and *waxy1*). The identified QTLs provide foundational targets for marker-assisted breeding. Few QTLs like *qHRD9*.1 (PVE = 14.18%) and *qTRP9*.1 (PVE = 10.69%) are prime candidates for improving hardness and tryptophan. These loci can be pyramided into elite lines using SSR markers; genomic selection could be used to optimize trait stacking. Future fine-mapping and functional studies will refine these regions, accelerating the development of high-yielding QPM with vitreous kernels and enhanced nutritional quality.

## 1 Introduction

Maize (*Zea mays* L.) is one of the world’s largest crops, serves as a staple crop for a billion people in Sub-Sahara Africa, Latin America, and Asian countries. Furthermore, besides being a staple food, it acts as a major vehicle for providing nutrients to humans and livestock (Shiferaw et al., 2011). Maize proteins generally lack sufficient levels of two important amino acids: lysine and tryptophan. The lysine level in maize kernel is typically 0.16-0.26%, while the tryptophan content is between 0.02-0.06%, which is less than half of the recommended dose specified for human nutrition (Mertz et al., 1964; Bjarnason and Vasal, 1992; Hossain et al., 2008; Vivek, 2008). The primary cause of the lack of lysine and tryptophan is mostly attributed to zein (prolamin fraction), a storage protein that makes up to 70% of all storage proteins (Gibbon and Larkins, 2005). This nature of maize kernel changed with the discovery of the natural *opaque (o2)* mutant, which led to the subsequent development of genotypes with increased lysine and tryptophan in maize endosperm (Mertz et al., 1964; Vasal, 2000; Gupta et al., 2009). The *o2* mutant has more accumulation of non-zein protein fractions, achieving the desired amino acid composition (Wang and Larkins, 2001). However, these mutant maize lines also have undesirable pleiotropic effects, such as soft and chalky kernels, and susceptibility to pests and diseases, which ultimately reduces yields (Gupta et al., 2013). To combat these effects, plant breeders introgressed quantitative trait loci (QTLs) known as *o2* modifiers (*Mo2*s) in *o2* mutant maize, developing what is now known as quality protein maize (QPM). This modifier-genetic system in the genetic background of the *o2* gene can transform the soft endosperm into a hard and vitreous endosperm without altering the increased protein content (Paez et al., 1969; Villegas et al., 1992; Or et al., 1993; Vivek, 2008; Babu et al., 2005; Krivanek et al., 2007; Pandey et al., 2016).

The inheritance of these *o2* modifiers has been proven to be highly complex but effective in improving the negative pleiotropic effects of *o2* genes (Vasal et al., 1980; Gibbon and Larkins, 2005). The modifier genes have been associated with elevated γ-zein, which renders the vitreousness in maize endosperm (Babu and Prasanna, 2014). Number of studies have aimed to comprehend the genetics behind the endosperm modifiers and their influence on grain quality in QPM with different genetic backgrounds. Lopes et al., 1995, using bulked segregant analysis (BSA), showed that chromosome 7 contains regions for endosperm modification. The first locus was found to encode the 27-kDa γ-zein and was located close to the centromere. The second locus was identified by using a limited set of restriction fragment length polymorphism (RFLP) markers, positioned near the telomeric ends of the long arm of chromosome 7 (Lopes et al., 1995). A number of studies have suggested that the γ-zeins play a major role in the regulation of endosperm modification (Geetha et al., 1991; Burnett and Larkins, 1999). Dannenhoffer et al. (1995) observed 27 kDa gamma-zeins to be involved in the development of the protein bodies responsible for the formation of the vitreous endosperm. Holding et al. (2008) examined individual F_2:3_ kernels for hardness and seed vitreousness and mapped seven *opaque2* modifier (*Opm*) QTLs on chromosomes 1, 5, 7, 9, and 10 using bulked sequencing analysis (BSA). Babu et al. (2015) utilized gene-based SSR markers of lysine and tryptophan and genomic SSRs on the F_2:3_ mapping population to identify five QTLs on chromosomes 5, 7, and 9. Later, it was shown that silencing or deletion of 27-kDa gamma zein expression abolished the formation of vitreous endosperm in QPM (Wu et al., 2010; Yuan et al., 2014). Zein proteins are stored in rough endoplasmic reticulum-retained protein bodies (PBs) in the maize endosperm (Wolf et al., 1967; Larkins and Dalby, 1975; Burr and Burr, 1976; Wu and Messing, 2010). The 27-kDa γ-zeins, along with 16-kDa γ-zein and 15-kDa β-zein, play an important role in the initiation and stabilization of protein bodies (PB) (Lending et al., 1992; Coleman et al., 1997). A significant QTL, namely *qy27*, was discovered to be a duplication of the 27-kDa γ-zein gene (Li et al., 2018). It was observed that this 15.26 kb duplicated segment regulated the elevated expression of the 27-kDa γ-zein gene in QPM. Furthermore, the correlation between the degree of modification and the level of 27-kDa γ-zein expression in different populations created by a cross between QPM and a starchy *o2* mutant suggested that the *qy27* controlling enhanced expression of 27-kDa gamma zein potentially houses an *o2* modifier (Li et al., 2018). However, still the total number of modifier genes affecting the endosperm modification is unknown.

To strengthen our knowledge of the genetic mechanism of the modifiers, we analyzed two different QPM lines, DQL 2104–1 and DQL 2034, with significantly varying kernel opacity, kernel hardness, and tryptophan content. Crosses were made to develop the F_2:3_ population for phenotypic and genotypic analysis. This study is distinct from earlier reports as it focuses on the features of kernel opacity, hardness, and amino acids in a population derived from the *opaque2* inbred lines. The objective of the present study is to identify genomic regions linked with endosperm modifiers affecting kernel opacity, tryptophan, and hardness that could be further utilized for the identification of key genes responsible for increased amino acid content and vitreous kernels and the development of better-performing QPM lines.

## 2 Materials and methods

### 2.1 Plant material

Two inbred lines, DQL 2104–1 and DQL 2034, significantly differing for kernel opacity, kernel hardness, and tryptophan content were crossed during Kharif 2021 ta Ladhowal Fields, ICAR-Indian Institute of Maize Research, Ludhiana, to generate F_1_ seeds (Anonymous, 2021). Approximately 350 F_1_ seeds were sown at the Agricultural Research Station (ARS), Peddapuram, Andhra Pradesh, during *Rabi* 2021. However, a reduced germination rate by ~40.59% was observed, yielding 138 F_2_ plants. These F_2_ individuals were self-pollinated at Ladhowal Farm in the *Spring* 2022 to produce F_2:3_ seeds. Due to heavy rainfall and waterlogging at the flowering stage, the seed set was impacted, resulting in only 109 F_2:3_ ears with >= 30 seeds per cob. The harvested F_2:3_ ears were used to examine kernel opacity, hardness, and tryptophan content. All the seeds were thoroughly dried and stored at 4°C.

### 2.2 Measurement of kernel opacity

The kernel opacity of F_2:3_ seeds was recorded using the lightbox (Bjarnason and Vasal, 1992) in three replications. The light box is used to differentiate between *opaque2* and normal grains based on their endosperm textures. The distorted three-dimensional structure of the kernel endosperm shows opaque seeds, whereas normal seeds are vitreous under the lightbox screening. For the screening, 10 randomly selected seeds per replication were examined. A total of thirty seeds were uniformly spread on the lightbox screen in such a way that the germ (embryo) part was facing down. The degree of opaqueness of seeds was calculated with the formula:


$$\text{Degree of opaqueness}=\left[\left({\text{N}}_{100}\times 100\right)+\left({\text{N}}_{75}\times 75\right)+\left({\text{N}}_{50}\times 50\right)+\left({\text{N}}_{25}\times 25\right)+\left({\text{N}}_{0}\times 0\right)\right]/100$$

Where, N_100_, N_75_, N_50_, N_25_, and N_0_ are the numbers of seeds with 100%, 75%, 50%, 25%, and 0% opacity, respectively (Hossain et al., 2008; Sarika et al., 2018).

### 2.3 Measurement of kernel hardness

Three seeds per replication were randomly selected and evaluated for kernel hardness by using a digital portable kernel hardness tester. The hardness was measured at a grain moisture content of approximately 14%. The kernels were placed in the chamber with the germ side up on a plate, and the crushing strength of individual seeds. To minimize the error, care was taken by placing all the seeds in the same direction. A test load of 5 to 500 N was applied, and breaking force was measured using the device’s frequency. The first peak force (N, Newton) in the force deformation curve was defined as kernel hardness (Zunjare et al., 2015).

### 2.4 Tryptophan estimation

The tryptophan content was estimated according to the colorimetric method (Hernandez and Bates, 1969). Ten seeds per genotype were randomly selected and were kept in distilled water overnight. The pericarp layer and germ layer were removed the following morning, and the seeds were left to dry in a hot air oven. The dried seeds were grounded with the help of a maize seed grinder, followed by defatting for 3 days (72 hours). The defatted samples (100 mg) were subjected to protein digestion (adding 4 mL of papain solution) and kept in an incubator at 65°C overnight. The following day, samples were processed using Hernandez and Bates, 1969. The standard curve for the tryptophan was taken using a standard tryptophan solution of known concentration against a blank at 545 nm. Thereafter, the factor was calculated using the standard curve to calculate percent tryptophan.

### 2.5 DNA extraction

Leaf samples were collected from all 138 F_2_ plants in the same season (*Rabi* 2021) - 30 days after germination (plant height: 15–20 cm) and were immediately stored at -80°C. Genomic DNA was extracted in two replicates per sample using the CTAB method (Murray and Thompson, 1980). DNA pellet was suspended in TE buffer (pH 8.0), and treated with RNase (10μg/mL) at 37°C for 35–40 minutes to remove RNA contamination. Purified DNA samples (including replication) were stored at -20°C in a 1.5 mL microcentrifuge tube. DNA quality was assessed by the intensity of DNA bands on a 0.8% agarose gel, while quantity was measured using a nanodrop spectrophotometer (ND 2000).

### 2.6 SSR marker selection and genotyping

A total of 496 Simple Sequence Repeats (SSR) markers, uniformly spread across all chromosomes, were used for parental polymorphism. Of these, 141 markers (28.43%) showed polymorphism and were selected for genotyping. From the original 138 F_2_ plants, 109 individuals were chosen for further analysis based on the F_2:3_ progeny available (genotypes having at least 30 seeds per cob).

### 2.7 PCR amplification and electrophoresis

Polymerase chain reaction (PCR) was carried out in a 10 μL reaction volume containing 1.5 μL template DNA, 1 μL forward and reverse primer each, 5 μL TaKaRa™ master mix (2X), and 1.5 μL nuclease-free water (NFW) in an Eppendorf MasterCycler™. The amplification conditions of the PCR reaction are as follows: first, initial denaturation at 95°C for 5 minutes, then 35 cycles of denaturation at 94°C for 40 seconds, annealing (temperature as per primer) for 40 seconds, extension at 72°C for 40 seconds and a final extension at 72°C for 8 minutes. The amplified products were resolved on 4% agarose gel stained with ethidium bromide and visualized under UV light.

### 2.8 Statistical analysis

The chi-square (χ^2^) test was used to determine deviations of observed genotypic ratios from the expected Mendelian segregation (1:2:1 for the F_2_ population) (Arora et al., 2021). Markers showing significant distortion (p<0.05) were eliminated from the further analysis.

### 2.9 Linkage map and QTL analysis

From the initial 141 polymorphic SSR markers (www.maizegdb.org), 25 markers showing significant segregation distortion (χ^2^) were removed, resulting in 116 informative markers. These markers were used to construct a genetic linkage map for the F_2_ population derived from the cross between DQL 2104–1 and DQL 2034. Linkage analysis was conducted using QTL IciMapping software version 4.2 (Wang et al., 2016) with a logarithm of odds (LOD) threshold of 3.0 for grouping and ordering markers. The Kosambi function was used to measure the map distance and was expressed in centimorgans (cM) (Kosambi, 2016).

## 3 Results

### 3.1 Phenotypic evaluation

**Figure 1:**
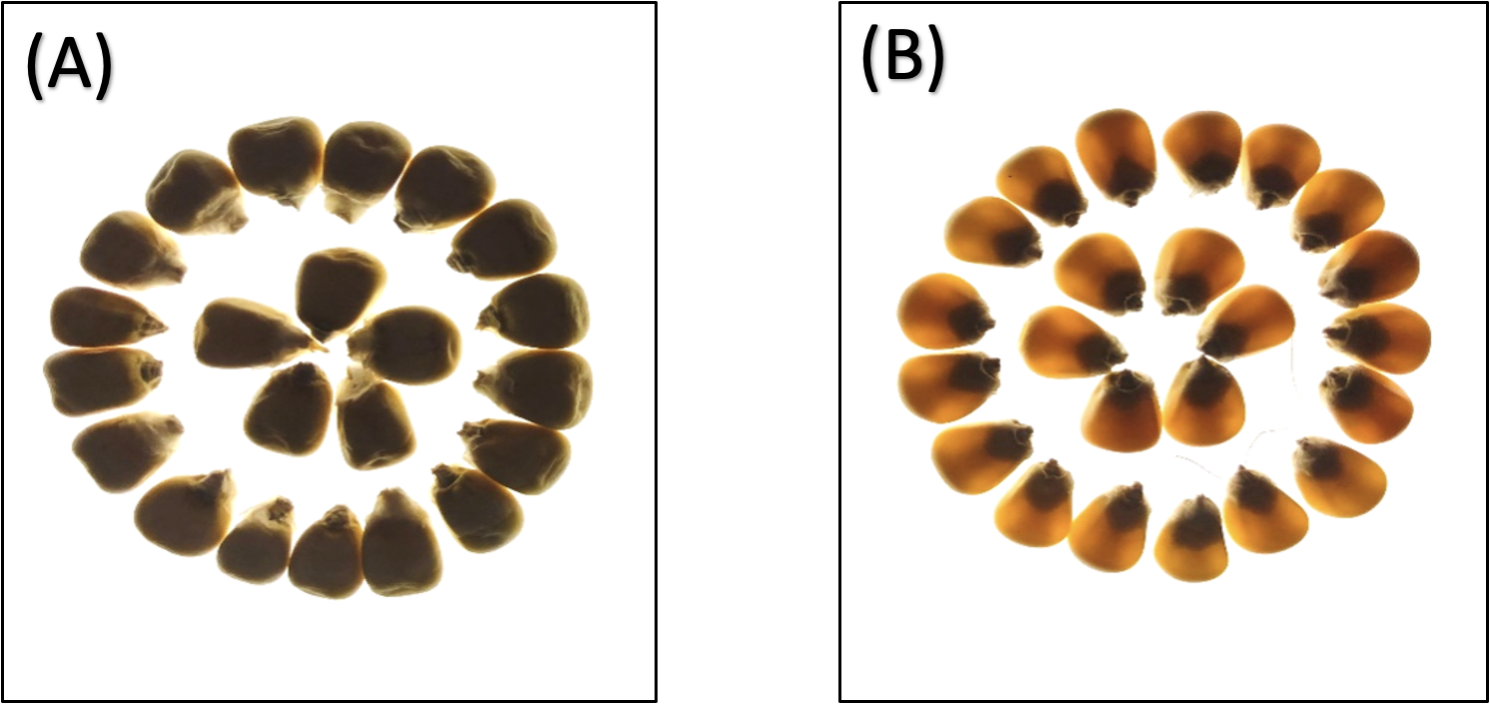
Light box testing of parents seeds: **(A)** Parent 1 – DQL2104-1 - 100% kernel opacity, **(B)** Parent 2 – DQL2034 - 25% kernel opacity.

**Figure 2:**
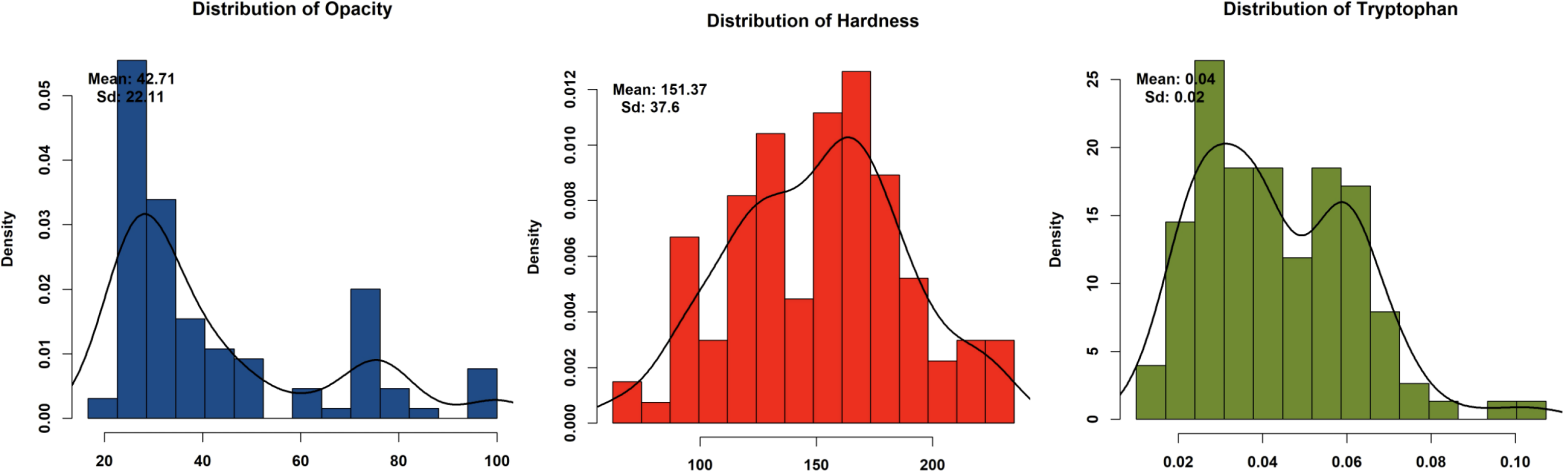
Graphical representation of density histogram of F_2:3_ population for kernel opacity, kernel hardness, and tryptophan content.

Phenotypic analysis of F_2:3_ progeny shows significant variation in three key endosperm traits: kernel opacity, hardness, and tryptophan content were estimated in F_2:3_ seeds (**Table 1**). The parent lines showed contrasting traits: DQL 2104–1 was fully opaque (100%) with lower hardness (Hardness: 95.83 N), while parent DQL 203 showed reduced kernel opacity (25%) and higher hardness of 163.17 N. The lightbox testing of parents and population is shown in **Figure 1**. The F_2:3_ population exhibited a broad range for kernel opacity (16.67 -100%), hardness (62.50-235.13 N), and tryptophan content (0.01-0.11%), demonstrating transgressive segregation (**Figure 2**). A negative relationship between opacity and hardness was observed, with seeds having lower opacity observed to have hard endosperm. The average tryptophan content was 0.081% in DQL 2104-1, 0.054% in DQL 2034, and 0.044% in the F_2:3_ population. The data about the kernel opacity, hardness, and tryptophan content of F_2:3_ population seeds is given in **Supplementary Table S1**.

**Table 1:** Descriptive statistics of the kernel opacity, hardness, and tryptophan content of the quality protein maize F_2:3_ mapping population.

Statistic	Kernel Opacity	Hardness	Tryptophan
Minimum	16.67%	62.50 N	0.01%
Maximum	100%	235.13 N	0.11%
Mean	42.71%	151.37 N	0.04%
Standard deviation	22.11	37.6	0.01
Co-efficient of variation	51.77%	25.01%	43.52%
Skewness	1.17	-0.02	0.64
Kurtosis	0.18	-0.41	-0.02

### 3.2 Correlation among the traits

**Figure 3:**
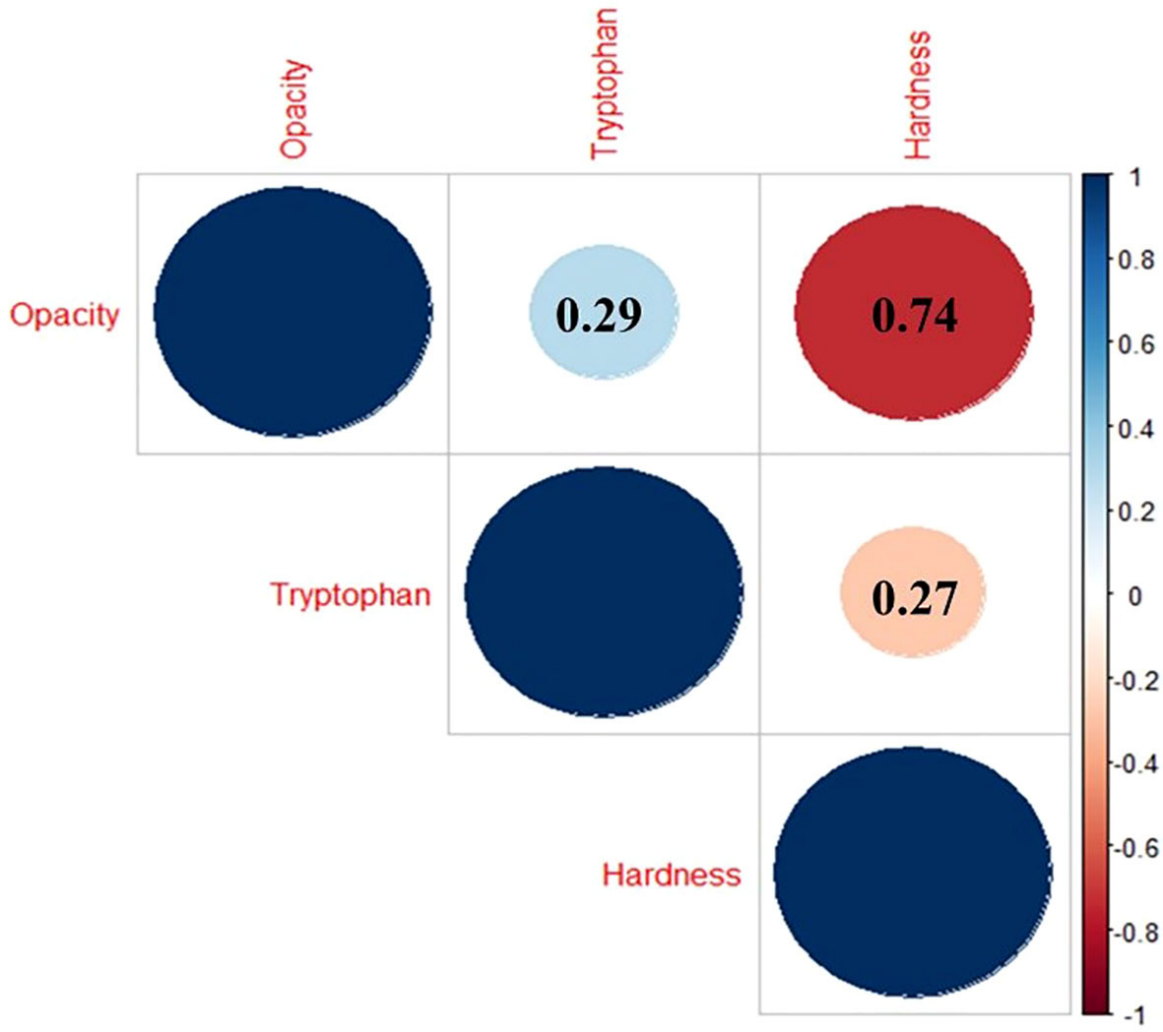
Correlation matrix between kernel opacity, kernel hardness, and tryptophan content (The color represents the direction of the correlation: the blue gradient indicates positive correlations, while the red gradient signifies negative correlations. The size of the circle indicated magnitude of the correlation within the matrix.).

The correlation analysis showed a significant correlation among traits (**Figure 3**). A weak but positive correlation (r = 0.29) was observed between tryptophan and opacity, indicating that an increase in kernel opacity is associated with an increase in tryptophan content. However, 25 genotypes (22.9%) do not follow the trend, exhibiting below-average opacity (<42.71%) coupled with higher tryptophan content (>0.045%), which might imply the role of genetic modifiers. Strong negative correlations were observed between kernel opacity and kernel hardness (r = -0.74) and kernel hardness and tryptophan content (r = -0.27), highlighting the inverse relationship between endosperm density and nutritional quality.

### 3.3 Genotyping and linkage map analysis

#### 3.3.1 *opaque2* gene confirmation in parental lines

**Figure 4:**
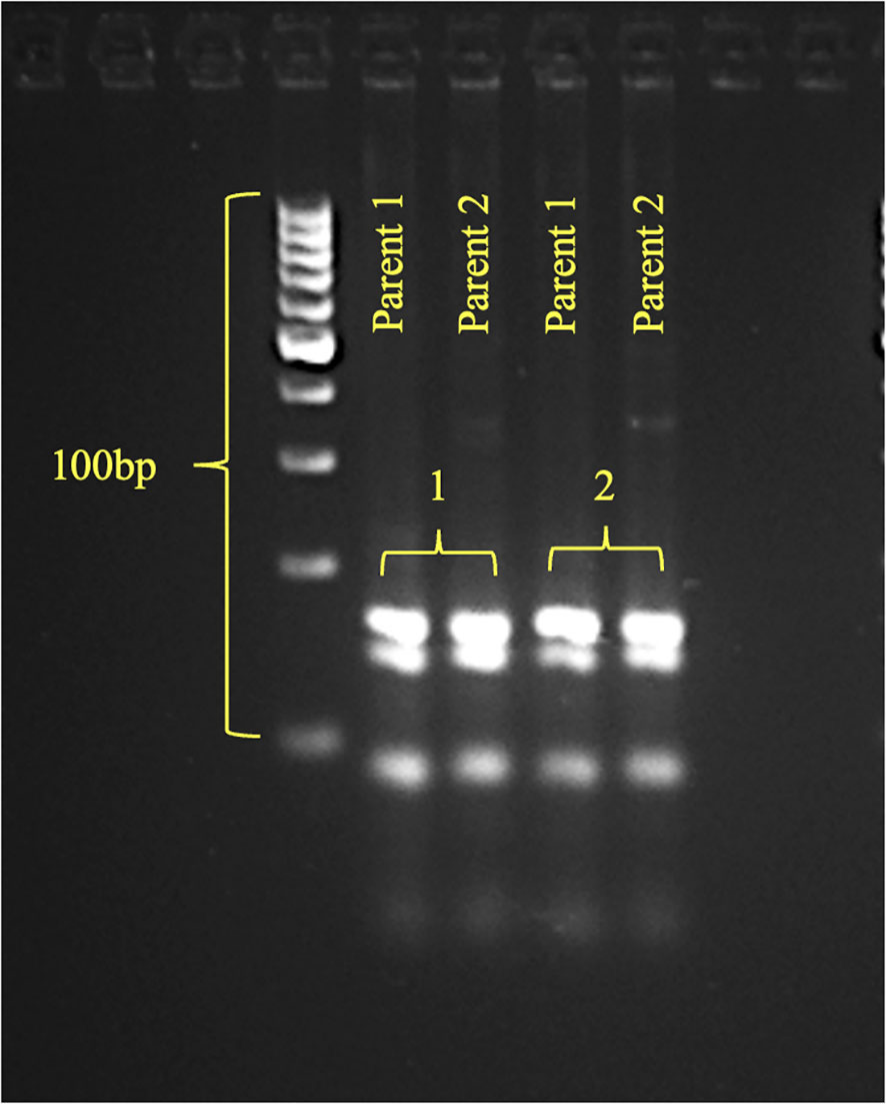
The gel pattern of parents (Parent 1 - DQL2104-1; Parent 2 - DQL2034) using *opaque2* SSR markers (1-phi057, 2-umc1066).

First, to validate the genetic basis of phenotypic divergence, both parents were screened with *opaque2-*specific co-dominate SSR markers, *i.e., umc1066* (bin 7.01) and *phi057* (bin 7.01) (Magulama and Sales, 2009). Monomorphic banding pattern for both markers confirmed the presence of the *o2* allele in both DQL 2104–1 and DQL 2034 (**Figure 4**). This result confirms that differences in kernel opacity, hardness, and tryptophan content between the parents are independent of *o2* presence, implicating additional genetic factor like modifiers, might be responsible for modulating these traits.

#### 3.3.2 Identification of polymorphic markers

**Figure 5:**
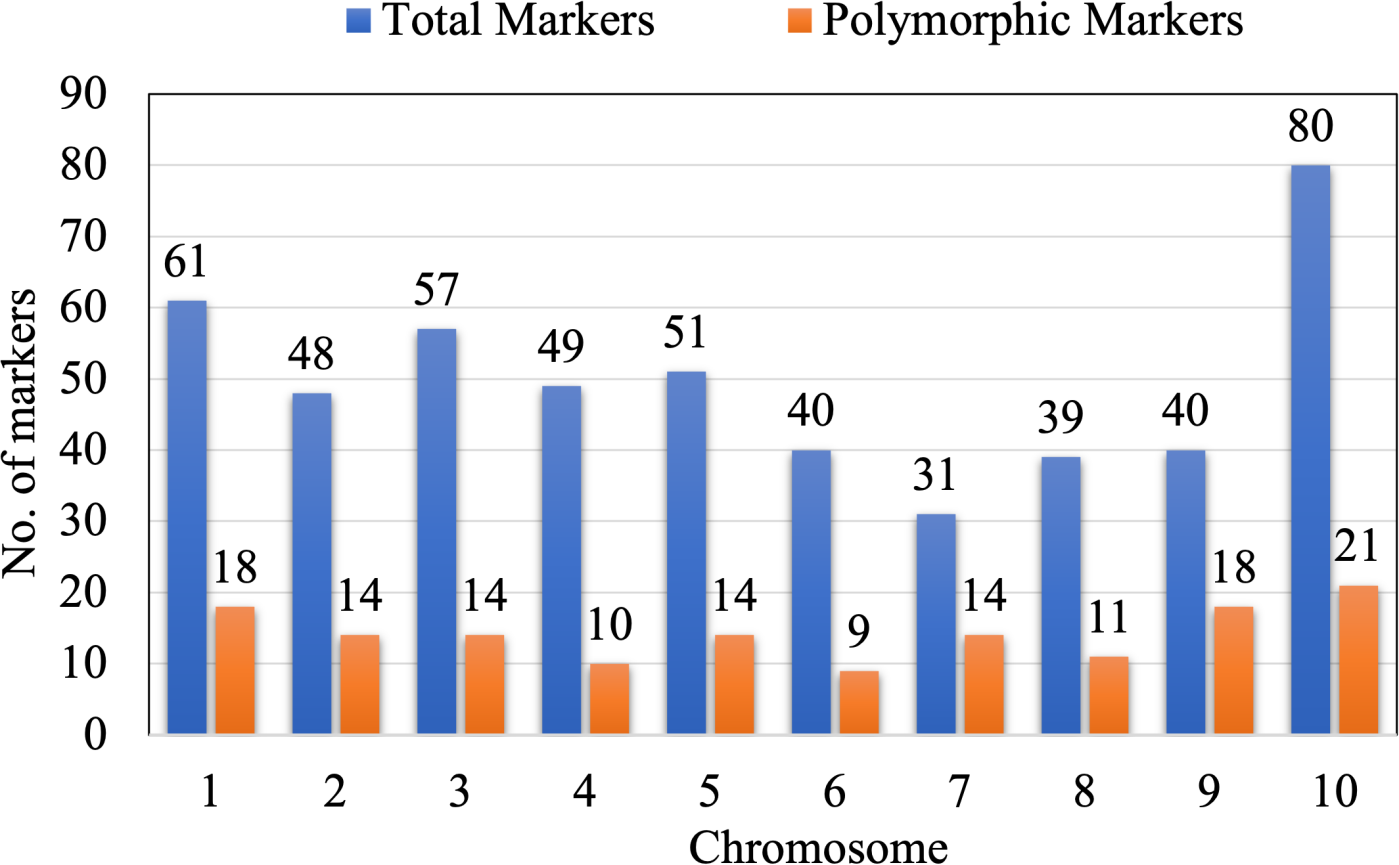
Graphical representation of total markers and polymorphic markers per chromosome of maize.

To identify genomic regions linked to endosperm modifiers and parents, DQL 2104–1 and DQL 2034 were screened using a total of 496 SSR loci, covering all ten maize chromosomes. Of these, 141 markers (28.43%) exhibited polymorphism and were selected for genotyping 109 F_2_ individuals (**Figure 5**). Chromosome 7 showed the highest polymorphism, with fourteen out of thirty-one (45.16%) markers and chromosome 4 showed the lowest (20.41%) with ten out of forty-nine markers.

#### 3.3.3 Chi-square (χ^2^) test for segregation distortion

To determine the segregation distortion of the marker loci, chi-square (*χ^2^*) was performed. Out of the total 141 SSR markers, twenty-five markers (17.73%) were removed from the analysis due to distorted chi-square values, resulting in 116 high-confidence polymorphic SSR marker loci spread across all the ten chromosomes (**Figure 5**). These markers were used to construct a linkage map with a total length of 2417.01 cM and an average distance of 20.66 cM between markers using IciMapping Version 4.2 software (Wang et al., 2016). Chromosome one was the longest linkage group (475.52 cM), while chromosome 6 was the shortest (155.33 cM). The information on the polymorphic primers, including their bin location, sequence, and annealing temperature, is summarized in **Supplementary Table S2**.

### 3.4 QTL analysis

**Figure 6:**
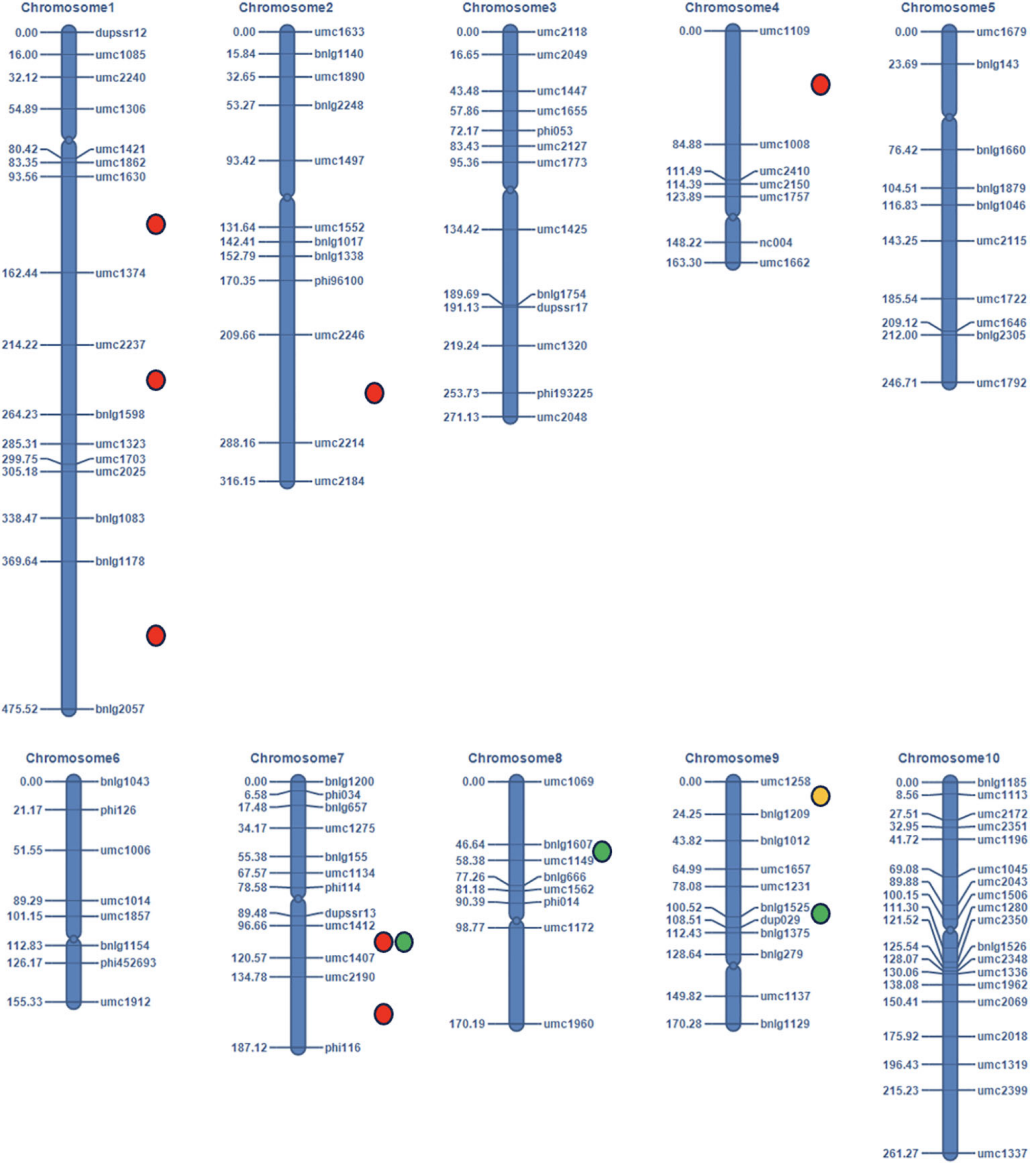
The linkage map (in cM) and location of the QTLs identified in the F_2:3_ mapping population of QPM maize using 116 polymorphic SSR markers (Colored circles are used to represent the QTLs for different traits: Red dots represent for kernel opacity, green for hardness, and yellow for tryptophan content).

The QTL mapping analysis was done using IciMapping V4.2 software (Wang et al., 2016). Inclusive composite interval mapping (ICIM) based on kernel opacity, hardness, and tryptophan content of 109 F_2:3_ individuals and genotypic data of 116 SSR markers was performed. Based on the analysis, we identified eleven QTLs on chromosomes 1, 2, 4, 7, 8, and 9 for all the traits (**Figure 6**). Seven minor QTLs were detected for kernel opacity on chromosomes 1, 2, 4, and 7, explaining a total phenotypic variance (PVE) of 20.76% (**Table 2**). Out of all seven, three QTLs on chromosome 1 for kernel opacity were detected between loci *umc1630* and *umc1374* with a LOD of 3.25, *umc2237* and *bnlg1598* with a LOD of 3.42, and *bnlg1178* and *bnlg2057* with a LOD 6.21 comprising total PVE of 9.81%. One QTL each on chromosomes 2 and 4 was identified for kernel opacity between marker loci *umc2246* and *umc2214* with an LOD value of 5.12 and between *umc1109* and *umc1008* with an LOD value of 3.97, respectively. The remaining two QTLs for kernel opacity were detected on chromosome 7 between marker loci *umc1412* and *umc1407* with an LOD value of 4.06 and a PVE value of 1.16% and between *umc2190* and *phi116* with an LOD value of 5.89 and a PVE value of 3.31%. A major QTL for tryptophan was mapped to chromosome 9, with a PVE of 10.69% and an LOD of 2.53, which is the second highest among all the QTLs identified. Three QTLs for kernel hardness were observed on chromosomes 7, 8, and 9. The QTL between the loci *bnlg1525* and *dup029* on chromosome 9 had a LOD value of 5 and a PVE value of 14.18%, which is the highest among all QTLs reported here and showed an additive effect contributed by DQL 2034. The QTL for kernel opacity with the LOD of 4.06 on chromosome 7 between the loci *umc1412* and *umc1407* coincides with the QTL for kernel hardness, indicating its pleiotropic nature. Another QTL for kernel hardness was detected on chromosome 8 between marker loci *bnlg1607* and *umc1149*, with an LOD of 2.99 and a PVE of 8.43% (**Table 2**). All the QTLs having higher LOD value (threshold value = 3) shows robust effect.

**Table 2:** The details of the identified QTLs for all three kernel traits with their LOD score, phenotypic variance (R^2^), additive effect, and dominance effect.

QTL name	Trait name	Chromosome	QTL peak	Markers	LOD	R^2^ (%)	Additive	Dominance
*qOPC1.1*	Opacity	1	128	umc1630-umc1374	3.24	3.25	-15.6854	-28.4575
*qOPC1.2*	Opacity	1	242	umc2237-bnlg1598	3.42	3.09	-14.2878	-29.8053
*qOPC1.3*	Opacity	1	422	bnlg1178-bnlg2057	6.21	3.47	16.6192	-29.2001
*qOPC2.1*	Opacity	2	246	umc2246-umc2214	5.12	3.17	21.3252	-17.6985
*qOPC4.1*	Opacity	4	63	umc1109-umc1008	3.97	3.31	11.1171	-34.8241
*qOPC7.1*	Opacity	7	117	umc1412-umc1407	4.06	1.16	14.4041	-5.998
*qOPC7.2*	Opacity	7	157	umc2190-phi116	5.89	3.31	15.3601	-28.7331
*qTRP9.1*	Tryptophan	9	0	umc1258-bnlg1209	2.53	10.69	0.0002	-0.012
*qHRD7.1*	Hardness	7	120	umc1412-umc1407	3.65	9.47	-17.9033	10.0577
*qHRD8.1*	Hardness	8	50	bnlg1607-umc1149	2.99	8.43	1.5087	23.6725
*qHRD9.1*	Hardness	9	103	bnlg1525-dup029	5.00	14.18	-21.6089	8.4449

## 4 Discussion

The nature and position of endosperm modifiers are very complex in most of the QPM germplasm, and it has been observed that *opaque2* modifiers often have an impact on several characteristics, including amino acids, kernel opacity, density, hardness, and vitreousness (Vasal et al., 1980; Gutierrez-Rojas et al., 2008; Holding et al., 2011). While *opaque2* (*o2*) mutations elevate lysine and tryptophan by suppressing α- zein synthesis, modifier genes restore kernel hardness without compromising nutritional quality. These endosperm modifiers are present along the ten chromosomes of maize, with dosage effects and cytoplasmic effects, adding to its complexity (Babu et al., 2015). The main objective of this study was to identify the genomic regions influencing the *o2* modifiers for three major traits, *viz.*, kernel opacity, kernel hardness, and tryptophan content in an F_2:3_ mapping population derived from QPM inbreds DQL 2104-1 (high opacity, soft kernels) and DQL 2034 (low opacity, hard kernels. Kernel opacity was examined with the help of a lightbox, which is a reliable method used to differentiate individual kernels based on different degrees of modification. The seeds having 100% opacity resulted in a completely black, opaque region, whereas normal seeds did not show any opaqueness. The seeds were characterized depending on the degree of opaqueness at 100%, 75%, 50%, 25%, and 0%. The second trait, *i.e.*, kernel hardness, was analyzed by the digital portable tablet hardness tester for 109 genotypes of the F_2:3_ population. About 52.29% of F_2:3_ genotypes were observed to have more hardness than the average. The third trait, tryptophan content, was estimated using a biochemical method, and it was observed that the 109 F_2:3_ population differed significantly.

A positive correlation (*r = 0.29*) between kernel opacity and tryptophan content was observed. The major storage proteins in maize are known as zeins, which are structurally divided into three types – alpha (α), beta (β), and gamma (γ) zeins. It has been studied that the *o2* mutation is responsible for the downregulation of α-zein proteins, which results in opaque endosperm (Hunter et al., 2002). Other studies reported that mutations in the transcriptional activator of the *o2* gene cause the concentrations of lysine and tryptophan to double (Henry et al., 2005; Maqbool et al., 2021). Conversely, the negative correlation between opacity and hardness was observed with *r = - 0.74*, underscoring the trade-off between nutritional quality and kernel density. This has been supported by numerous studies that have demonstrated a relationship between the phenotype of the kernel and the deposition of protein bodies during endosperm development (Tsai et al., 1978; Woo et al., 2001). Our result in this study is in agreement with previous studies suggesting a positive correlation between amino acid content and degree of opaqueness (Scott et al., 2004; Gutiérrez-Rojas et al., 2010; Babu et al., 2015) and a negative correlation between hardness and opacity (Holding et al., 2008; Kaur et al., 2020). However, a small number of genotypes were found to deviate from this pattern, which could be accounted for by the impact of endosperm modifiers in the population. The *o2* mutation usually decreases the α-zein protein in the kernel, which results in a soft and opaque kernel with a high amino acid content. However recent research has revealed that a 15.26 kb duplicated section (*qy27*) at the 27 kDa locus of γ-zein is a key modifier of QTL that induces endosperm hardness and increases protein expression (Liu et al., 2016). Due to the modifier genes, the soft and dull kernels get converted into the hard and vitreous type without affecting the amino acid content, hence explaining the few deviations in our study pattern.

### 4.1 QTL analysis for kernel opacity

The genetic complexity of kernel opacity in maize is emphasized by the identification of seven QTLs acting as modifiers through their influence on starch metabolism, storage protein synthesis, and endosperm architecture. These are distributed across chromosomes 1, 2, 4, and 7 and are overlapped with functionally validated genes for kernel texture to nutritional quality. The QTL *qOPC1.1* on chromosome 1 is correlated with the *OHP1* gene (opaque2 heterodimerizing protein 1), a bZIP-type transcriptional factor (TF). *OHP1* was first identified as a paralog of the *O2*-box motif in the promoters of zein genes, playing a critical role in regulating the 27-kDa gamma zein genes (Pysh et al., 1993; Pysh and Schmidt, 1996; Yang et al., 2016; Li and Song, 2020). Studies have shown that *OHPs* directly impact tryptophan content by synergistically coactivating the expression of zein genes, and its silencing reduces 27-kDa gamma-zein proteins (Zhang et al., 2015; Yang et al., 2016; Zhan et al., 2018). Therefore, *OHP* is crucial for 27 kDa-zein transcription, which promotes the concentration of amino acids in QPM lines and facilitates the synthesis of protein bodies and the efficient accumulation of zein storage protein (Li et al., 2018). This locus plays a dual role in maintaining vitreous endosperm while enhancing amino acid profiles. The QTL on chromosome 2, *qOPC1.2* aligns with earlier studies reporting loci in bin 1.06 (*umc1335*) that influence lysine and kernel opacity/vitreousness (Gutierrez-Rojas et al., 2010; Salazar-Salas et al., 2014). The QTLs having lower PVE value, despite a robust LOD score may not have major affect but may have synergetic effect with other loci.

The identification of *qOPC2.1* on chromosome 2 is a novel finding, as no QTL for kernel opacity has previously been reported on chromosome 2. This discovery highlights the complex nature of genetic modifiers and suggests allelic diversity in current maize germplasm unmasked this locus. This locus overlaps with three functionally characterized genes - namely, *floury1* (*fl1*), *opaque11* (*o11*), and *mucronate 1* (*Mc1*) were found within the genomic interval of *qOPC2.1*. Each gene plays a distinct role in endosperm development and composition. The *opaque11* (*o11)* gene encodes for the basic helix-loop-helix (bHLH) transcription factor unique to cereal endosperm. Mutation in *o11* results in smaller, opaque endosperm that contains less starch and protein. Further, this transcription factor coordinates and regulates cell development and storage metabolism and has a positive role in starch accumulation (Feng et al., 2018). The *mucronate* (*Mc*) gene, another candidate within this interval, is a dominant mutation that reduces the amount of zein protein synthesis (a major class of maize storage protein) (Zhang and Boston, 1992; Kim et al., 2006). This reduction in zein protein disturbs the formation of protein bodies (PBs). The antagonistic relationship between these genes exemplifies how modifier loci balance starch and protein deposition. Previous studies have also demonstrated that the interaction of PBs, cytoplasm, and starch granules is important for the development of opaque endosperm (Wolf et al., 1967; Holding, 2014; Zhang et al., 2018).

A single QTL on chromosome 4, *qOPC4*.1, coincides with three important genes - *floury2 (fl2), floury4 (fl4)*, and *Opaque1 (O1)*. Semi-dominate mutation involving the *fl2* and *fl4* genes results in the formation of soft and starchy maize endosperm due to the accumulation of novel 24-kDa α-zein proteins, which disrupts the PB formation and starch-protein interactions (Lopes et al., 1994; Coleman et al., 1995). The *O1*, encoding a Myosin XI Motor Protein, regulates the endoplasmic reticulum (ER), influencing PB formation and endosperm texture. Mutation in *O1* disrupts the ER and leads to the formation of PB formation and opaque kernels (Wang et al., 2012). The association of these loci with *qOPC4.1* emphasizes its role as a key modifier of kernel opacity.

On chromosome 7, which was previously reported as a hub for kernel modifiers (Lopes et al., 1995; Holding et al., 2008; Babu et al., 2015), two additional loci, namely, *qOPC7.1* and *qOPC7.2*, were mapped. It has been discovered that this QTL is pleiotropic, influencing both kernel opacity and kernel hardness. Also, this locus coincided with genomic regions reported in earlier studies (Holding et al., 2008), where markers like *umc2190* were linked to kernel vitreousness. The second locus *qOPC7.2*, showed additive and dominant effects which aligned with previous studies (Holding et al., 2008).

These findings highlight the genetic complexity of kernel opacity but also suggest the direct impact of these loci on amino acid enrichment and starch-protein balance. While the study successfully identified QTLs associated with kernel opacity, the broad confidence interval (> 50 cM) observed for several QTLs reflects the limitation of low marker density and recombination events in the F_2_ population. To resolve this, further studies of fine mapping using advanced populations and high-density markers are required to narrow down intervals, enabling precise identification of candidate genes. These loci can be incorporated into predictive models to exploit additive effects for kernel opacity.

### 4.2 QTL analysis for kernel hardness

Three QTLs, *viz., qHRD7.1*, *qHRD8.1*, and *qHRD9.1*, were identified for kernel hardness on chromosomes 7, 8, and 9, respectively. The *qHRD7.1* co-localized with the kernel opacity QTL on chromosome 7, suggesting that this locus could have a pleiotropic effect, a shared genetic mechanism influencing both hardness and opacity. Therefore, it is a strategic target for breeders aiming to simultaneously improve kernel texture and nutritional traits. On chromosome 8, a novel QTL (*qHRD8.1*) coincides with *OHP3* (*O2*-heterodimerizing protein 3), another transcriptional regulator for zein genes. While previous QTL on chromosome 8 reported QTLs for texture, opacity, and vitreousness (Gutierrez-Rojas et al., 2010), *qHRD8.1* also has a pleiotropic effect for both hardness and opacity. The most impactful locus, *qHRD9.1*, on chromosome 9 has the highest PVE among all identified QTLs and exhibits a strong additive effect contributed by parent DQL2034.

### 4.3 QTL analysis for tryptophan content

One highly effective QTL was identified for tryptophan content on chromosome 9, explaining a phenotypic variance of 10.69%, which is the second highest among the QTLs. This locus coincides with the QTL identified in a previous study (Babu et al., 2015). The locus co-localized with the *waxy 1* (*Wx1*) gene, encoding for granule-bound starch synthase 1 (GBSS1), a key enzyme influencing both amino acid metabolism and starch composition (Babu et al., 2015). While *Wx1* is traditionally linked with amylose synthesis and vitreous endosperm formation (Gibbon et al., 2003), our findings suggest a dual role in influencing tryptophan levels. This aligns with the prior studies (Babu et al., 2015), where *Wx1* was linked to *opaque2* modifiers, further validating its importance in maize improvement.

The major QTLs in our investigation are identified on chromosomes 1, 7, and 9, in accordance to past studies (Holding et al., 2008; Babu et al., 2015), established that these chromosomes are the major hub of the endosperm modifiers. These loci, including *qHRD9.1* (chromosome 9, PVE = 14.18%) and *qTRP9.1* (chromosome 9, PVE = 10.69%), which align with earlier findings that highlight the importance of γ-zein genes and their regulators in protein body formation and starch-protein interactions (Geetha et al., 1991; Lopes and Larkins, 1995; Burnett and Larkins, 1999; Hunter et al., 2002; Adunola et al., 2017). Protein 27-kDa γ-zein has been shown to be crucial for the development of protein bodies and the formation of the protein network that encircles starch grains in the endosperm (Dannenhoffer et al., 1995; Gutiérrez-Rojas et al., 2010; Pandey et al., 2016). Furthermore, it has been noted that variations in the polypeptide levels of the enzymes responsible for starch production and the branching pattern of starch have been explained by variations in phenotypes (Gibbon et al., 2003).

While the major genes could be utilized in Marker-Assisted Selection (MAS) and gene pyramiding, the minor genes (with low PVE value) may have epistasis effects to enhance traits as these QTLs could synergistically enhance both kernel hardness and lysine/tryptophan levels. Despite small individual effects, these QTLs have potential in breeding program. Notably, three loci *qOPC7.1*, *qHRD7.1*, *qHRD8.1*) may have pleiotropic effects, emphasizing there shared role in genetic mechanisms between kernel texture and nutritional traits. These loci could be used in pyramiding in elite lines. Genomic Selection (GS) can be used to integrate these loci into predictive models, allowing breeders an ability to exploit additive genetic variance across the genome.

In this study, low recombination event and low marker density contributed to broad confidence interval in QTL regions associated with endosperm modifications and amino acids. The current findings advance our understanding of the regions governing the endosperm modifiers in quality protein maize. To enhance the efficiency of QTL mapping, further strategies like fine mapping, haplotype analysis, genome-wide association studies (GWAS) can be done to get more detail information of genetics of endosperm modifiers. Additionally functional validation of candidate genes gives deeper insight into genetic regulation of endosperm modifications. This will accelerates the development of improved QPM varieties with improved nutritional quality an agronomical performance compared to traditional breeding methods.

## Data Availability

The original contributions presented in the study are included in the article/**Supplementary Material**, further inquiries can be directed to the corresponding author/s.
